# Elevated root-zone P and nutrient concentration do not increase yield or cannabinoids in medical cannabis

**DOI:** 10.3389/fpls.2025.1433985

**Published:** 2025-02-20

**Authors:** Julie A. Hershkowitz, F. Mitchell Westmoreland, Bruce Bugbee

**Affiliations:** Crop Physiology Laboratory, Department of Plants, Soil, and Climate, Utah State University, Logan, UT, United States

**Keywords:** cannabis, phosphorus, electrical conductivity, cannabinoid, plant nutrition

## Abstract

Elevating nutrient input is thought to increase yield and cannabinoid concentration of medical cannabis, but increased legalization has heightened awareness of the environmental impact of overfertilization. Elevated levels of phosphorus (P) are of particular concern. Here we report the effects of increasing P above levels adequate for other crops (15, 30, 45, 60, or 90 mg per L) and the interactive effects of elevated P with elevated nutrient solution concentration (electrical conductivity; 2 and 4 mS per cm). We used closed-system hydroponics to continuously quantify rootzone nutrient concentrations. The concentration of P in leaf tissue doubled and flower P concentration increased 70% when the P input increased from 15 to 90 mg per L but there was no difference in yield or quality among treatments. Doubling nutrient input from 2 to 4 mS per cm increased nutrient accumulation in solution but did not significantly increase yield or quality. Reducing P in the refill solution from 90 to 15 mg per L reduced P in solution at harvest from 300 to less than 0.1 mg per L. Despite the low steady-state concentration of P in solution in the 15 mg per L treatment, there was no difference in yield or quality among treatments, regardless of the concentration of other elements. Despite the high nutrient concentrations in the rootzone solution there was no leaf necrosis or other visual effects among treatments. These data indicate cannabis tolerates high nutrient concentrations, but neither excessive P nor excessive fertilization improves yield or quality.

## 1 Introduction

There is a growing awareness of the environmental impacts of medical cannabis (*Cannabis sativa* L.) cultivation (Ashworth and Vizuete, 2017; Butsic and Brenner, 2016; Wilson et al., 2019; Zheng et al., 2021). Adequate nutrition is critical for optimal yield, but excessive fertilizer application, particularly phosphorus (P), is common in medical cannabis production. Some studies suggest medical cannabis may require higher nutrient inputs than other crops (Bevan et al., 2021), but numerous studies have reported negligible effects of increased nutrient supply on yield and quality (Anderson et al., 2021; Bevan et al., 2021; Saloner and Bernstein, 2022; Shiponi and Bernstein, 2021b; Westmoreland and Bugbee, 2022).

Optimal application rates of phosphorus (P) are well studied in field agriculture, yet this element is often applied to excess. A recently published book titled “*The Devil’s Element: Phosphorus and a World Out of Balance*” (Egan, 2023) provides a comprehensive review of the detrimental environmental effects of excess P in agricultural systems. Westmoreland and Bugbee (2022) reviewed the nutrient imbalances associated with excess P in both field and controlled environments.

Elevated P can interact with the concentration of other elements. In medical cannabis cultivation, nutrients are often elevated to induce osmotic stress, which has the potential to increase carbon partitioning to reproductive structures. Osmotic stress might also help to concentrate specialized metabolites in flowers and fruits. This osmotic stress is sometimes achieved by adding only macronutrients, but this can lead to nutrient imbalances, so it is also achieved by elevating all macro- and micronutrients. In field environments, precision water stress is usually achieved by regulated deficit irrigation, but this is extremely difficult to achieve in containerized production, especially with soilless media.

Several recent studies have examined the effects of elevated nutrient concentrations. High solution EC can induce phytotoxicity, and reduce yield (Machado and Serralheiro, 2017; Savvas and Adamidis, 1999), but cannabis may to be more tolerant of high fertilizer inputs than other crops. Baas and Wijnen (2023) reported no difference in yield between plants grown at an EC of 1.8 and 3 mS per cm but increasing EC to 12 mS per cm reduced yield by three-fold. Notably there was no visible phytotoxicity up to an EC of 12 mS per cm when EC was increased by increasing nutrient concentrations, but increasing EC with NaCl induced phytotoxicity at 9 mS per cm. Baas and Wijnen (2023) also reported no difference in THCeq concentration with ECs from 1.8 to 12 mS per cm.

Controlled environments facilitate rigorous monitoring and control of nutrient and water input and allow for precision fertilization. The nutrient input required to achieve a desired tissue concentration can be calculated from first principles. Sufficiency ranges of tissue nutrient concentrations have been described for most crops, including cannabis (Bryson and Mills, 2015; Kalinowski et al., 2020; Landis et al., 2019; Marschner, 2012). The principle of mass balance, which assumes all nutrients supplied to a system are taken up by the plant or remain in solution, can be used to optimize the efficiency of a nutrition program (Bugbee, 2004; Langenfeld et al., 2022).

Nutrient solution composition and concentration are important considerations. Electrical conductivity (EC; mS per cm) is a common metric to quantify solution concentration, but it does not describe the solution composition. EC is primarily determined by macronutrient (Ca, S, N, P) concentrations with micronutrients contributing less than 1%. Differences in nutrient uptake can lead to imbalances in the recirculating solution, which is exacerbated by excessive supply in the refill solution (Bugbee, 2004). Nutrients with active uptake (N, P, K, Mn) are depleted quickly, while nutrients with intermediate and passive uptake (Ca) tend to accumulate (Bugbee, 2004; Savvas and Adamidis, 1999; Savvas and Gizas, 2002). Such imbalances could induce ion precipitation, antagonism, or phytotoxicity (Bugbee, 2004; Kaya and Higgs, 2001). Active uptake may result in low solution EC, while tissue concentrations are within the optimal range for metabolic function (Bugbee, 2004; Langenfeld et al., 2022).

Luxury uptake of nutrients without a corresponding increase in yield has been observed in vegetative and flowering cannabis (Chapin et al., 1990; Saloner and Bernstein, 2020, 2022; Shiponi and Bernstein, 2021a, b; Westmoreland and Bugbee, 2022). Cannabis is particularly prone to accumulating P in flower tissue (Shiponi and Bernstein, 2021a, b; Veazie et al., 2021; Westmoreland and Bugbee, 2022). Massuela et al. (2023) observed that flower P was around 4 times higher than leaf P and Shiponi and Bernstein (2021b) reported that flower P accounted for 80% of total plant P. Westmoreland and Bugbee (2022) reported that P concentrations increased by 35% in leaves and 11% in flowers when the P input increased from 25 to 75 mg per L. Interestingly, two studies reported that tissue P increased with increasing P supply up to 60 mg per L, but was unaffected by further increases in P supply (Shiponi and Bernstein, 2021b; Massuela et al., 2023).

Our objective was to quantify the effects of nutrient solution concentration (EC; 2 and 4 mS per cm) and the interactive effects with P supply (15, 30, 45, 60, and 90 mg per L) on yield, quality, and nutrient partitioning of medical cannabis in closed system, deep-flow hydroponics.

## 2 Materials and methods

### 2.1 Plant material

Two trials were conducted in time. For each trial 50 cuttings of the high-CBD medical cannabis cultivar ‘T1’ (also called “Trump”) were collected from the same mother plant, treated with rooting hormone (Hormodin 2, Indol-3-butyric Acid 0.03%, OHP Inc), and rooted in course perlite for two weeks. The chemotype III cultivar ‘T1’ was selected because it has high yields in controlled environments and has been characterized in previous studies; Westmoreland et al., 2021; Westmoreland and Bugbee, 2022). Twenty-four rooted cuttings were selected for uniformity and transplanted into a 48 L (57.5 x 44.5 x 23.5 cm) aerated, deep-flow hydroponic tub. One day after transplant, plants were pinched to four nodes and grown vegetatively (18/6 h, light/dark) for seven days in a greenhouse. After seven days, plants were moved to a walk-in growth chamber with a reproductive photoperiod (12/12 h, light/dark) to begin treatments. Four plants were randomly assigned to one of six 48 L tubs with their respective nutrient solution treatments. The resulting plant density was 20 plants per m^2^ with equal-distant spacing of the plants in all treatments. After seven days of reproductive photoperiod, plants were pinched a second time to achieve two nodes per branch for a total of eight branches on each plant. Plants were harvested 56 days after the induction of reproductive growth.

### 2.2 Environment

White + red LEDs (Dragon Alpha, Scynce LED) provided a photosynthetic photon flux density (PPFD; 400 to 700 nm) of 923 ± 102 μmol·m^-2^·s^-1^ (mean ± standard deviation; daily light integral (DLI) of 40 ± 4 mol·m^-^²·d^-1^) at canopy height throughout the study. PPFD was measured every seven days with a handheld quantum sensor (MQ-500, Apogee Instruments Inc.) and lights were dimmed as plants grew. The air in the chamber was well mixed and the air temperature was spatially uniform among treatments at 26.1 ± 1.4/24.2 ± 1.2°C (day/night ± SD), measured with a shielded, fan aspirated thermistor (model ST-100, Apogee Instruments Inc.). Relative humidity (RH) was 60 ± 9/51 ± 7% (day/night ± SD) and the vapor pressure deficit (VPD) was 1.4 ± 0.3/1.5 ± 0.2 kPa (day/night ± SD) measured with a temperature and RH probe (model HMP45A, Campbell Scientific Inc.). Fans supplied airflow of about one m per s at the top of the canopy measured with a hot-wire anemometer (TSI Inc., model 8330). Environmental measurements were made every ten seconds and ten-minute averages were recorded by a datalogger (model CR1000X, Campbell Scientific Inc.). All of the environmental parameters were uniform and constant over the course of the study.

### 2.3 Nutrient solution treatments

Nutrient solutions were formulated using the mass-balance approach described by Langenfeld et al. (2022). Solutions were prepared using deionized (DI) water and reagent grade salts (Sigma Aldrich). The salts and the procedures used to make the solutions are described in Langenfeld et al. (2022).

During vegetative growth all plants received the same nutrient solution at an EC of 2 mS per cm. The composition of the nutrient solutions at high and low EC are shown in **Supplementary Tables 1** and **2**.

Nutrient solution treatments are reported as the EC of the refill solution (**Supplementary Table 1**). Individual treatments consisted of a nutrient solution concentration (2 or 4 mS per cm) and a P concentration (15, 30, 45, 60, 90 mg per L). The lowest P concentration reflects a commonly used fertigation concentration for crops in commercial greenhouse production. The highest rate is often used by medical cannabis growers and is recommended by some cannabis fertilizer manufacturers. Shiponi and Bernstein (2021b) and Westmoreland and Bugbee (2022) studied a similar range of rates, but only at a lower EC level. The high EC treatment was achieved by doubling the concentration of all nutrients except P. P was supplied as KH_2_PO_4_. Total P and additional K from KH_2_PO_4_ is summarized in **Supplementary Table 2**.

The low EC treatment represents an adequate but not excessive concentration of nutrients for rapid growth of crops in controlled environments (Langenfeld et al., 2022). The high EC concentration is often used by growers to reduce internode elongation.

Tub solutions were manually refilled daily to maintain a volume of 48 L. Prior to refill, EC and pH of the tub solution was measured with an EC (Dist. 4, Hanna Instruments) and pH meter. After refill, pH was measured and adjusted using HNO_3_ or KOH to maintain a pH between 5.8 and 6.2. The cumulative volume of refill solution was not statistically different among treatments.

### 2.4 Tissue and root-zone solution analysis

Leaf, flower, and solution samples were collected every seven days for element analysis. Three recently expanded fan-leaves and three grams (dry mass) of flower were collected from each plant within each treatment. Flower samples were collected from the upper inflorescences of each plant, inflorescence leaves were excluded. Tissue samples were washed with deionized water and dried at 80°C for 48 h then ground to a fine powder with a stainless-steel grinder. Solution samples were collected prior to refilling tub solutions. Leaf and flower sample from each plant within each tub was homogenized and analyzed as a single sample. All samples were submitted to the Utah State University Analytical Laboratory for mineral analysis using inductively coupled plasma-optical emission spectrometry (ICP-OES) (Thermo Scientific iCAP 6300 Spectrometer, Thermo Fisher Scientific Inc.). Solution N was analyzed using Flow Injection Analysis (QuikChem 8000, Lachat Instruments; method 10-107-04-1-C). Tissue N was determined using combustion analysis (Elementar VarioMax Cube, Elementar Americas Inc.). Tissue concentrations are reported on an oven-dry weight basis (mg per g).

### 2.5 Cannabinoid analysis

At harvest, flowers were collected from the upper inflorescences of each plant in each treatment for cannabinoid analysis. Samples were dried on a ventilated rack at 25°C and 30% RH for five days then ground to a fine powder using a stainless-steel grinder. Samples from each plant in a treatment were homogenized and analyzed as a single sample. Samples were analyzed by the Utah Department of Agriculture and Food (UDAF) Unified State Laboratory. Cannabinoid equivalents (CBD_eq_ and THC_eq_) were calculated following Westmoreland et al. (2021). Cannabinoid analytical procedures were as described in Westmoreland and Bugbee (2022).

### 2.6 Plant measurements and harvest

Plants were destructively harvested 56 days after starting treatments. Height was measured from the base of the stem to the apex of the dominant inflorescence. Plants were cut at the base of the stem just above the root ball. Leaves and flowers were mechanically stripped from stems using a bucker (High Performance Tabletop Bucker, Centurion Pro Solutions Ltd). Inflorescence leaves were separated from flowers with a trimmer (Tabletop Trimmer, Centurion Pro Solutions Ltd). Roots, stems, leaves, and flowers were dried in an oven at 80°C for 48 h. Flower yield (grams per m^2^) was calculated as the total oven-dried flower divided by the canopy area of each tub. Harvest index (HI) was calculated as the ratio of flower mass to total biomass (flowers, leaves, stems, and roots).

### 2.7 Experimental design and statistical analysis

The study was a randomized incomplete block design. There were three P levels and two EC levels in each block, resulting in six treatments. The study was repeated twice, resulting in two blocks in time. In the first study (block) the P levels were 30, 60 and 90 mg per L. Because there was no significant effect of P; in the second replicate study the P levels were 15, 30 and 45 mg per L. Each tub containing four plants was treated as an experimental unit. Data were normalized (referenced) to 30 mg per L P between studies and analyzed using regression. All responses fit a simple linear model, so more complex, quadratic models were not used. P was treated as a continuous variable, and EC was treated as a factor. Effects were considered significant at α = 0.05. All statistical analysis were performed in RStudio (R statistical software, version 4.1.0.).

## 3 Results

### 3.1 Yield and cannabinoid concentration

There were no visual differences in plant health among treatments. Dry flower yield was not significantly affected by P supply (p = 0.95) or solution EC (p = 0.22; **Supplementary Figure 1**). The average dry flower yield across all treatments was 640 ± 88 g·m^-2^ (mean ± SD). There was no effect of P supply (p *=* 0.48) or solution EC (p *=* 0.12) on harvest index (HI) (**Supplementary Figure 2**). The average HI across all treatments was 48 ± 4%. There was no significant effect of solution EC or P on concentrations of CBD_eq_, THC_eq_, or the ratio of CBD_eq_ to THC_eq_. Across all treatments, CBD_eq_ and THC_eq_ were 13.55% ± 0.85 and 0.59% ± 0.04, respectively (**Supplementary Figure 3A, B**). The ratio of CBD_eq_ to THC_eq_ was 22.9 ± 1.3 across treatments (p = 0.45 (EC); p = 0.84 (P)).

### 3.2 Root-zone solution nutrient concentrations over time

**Figure 1:**
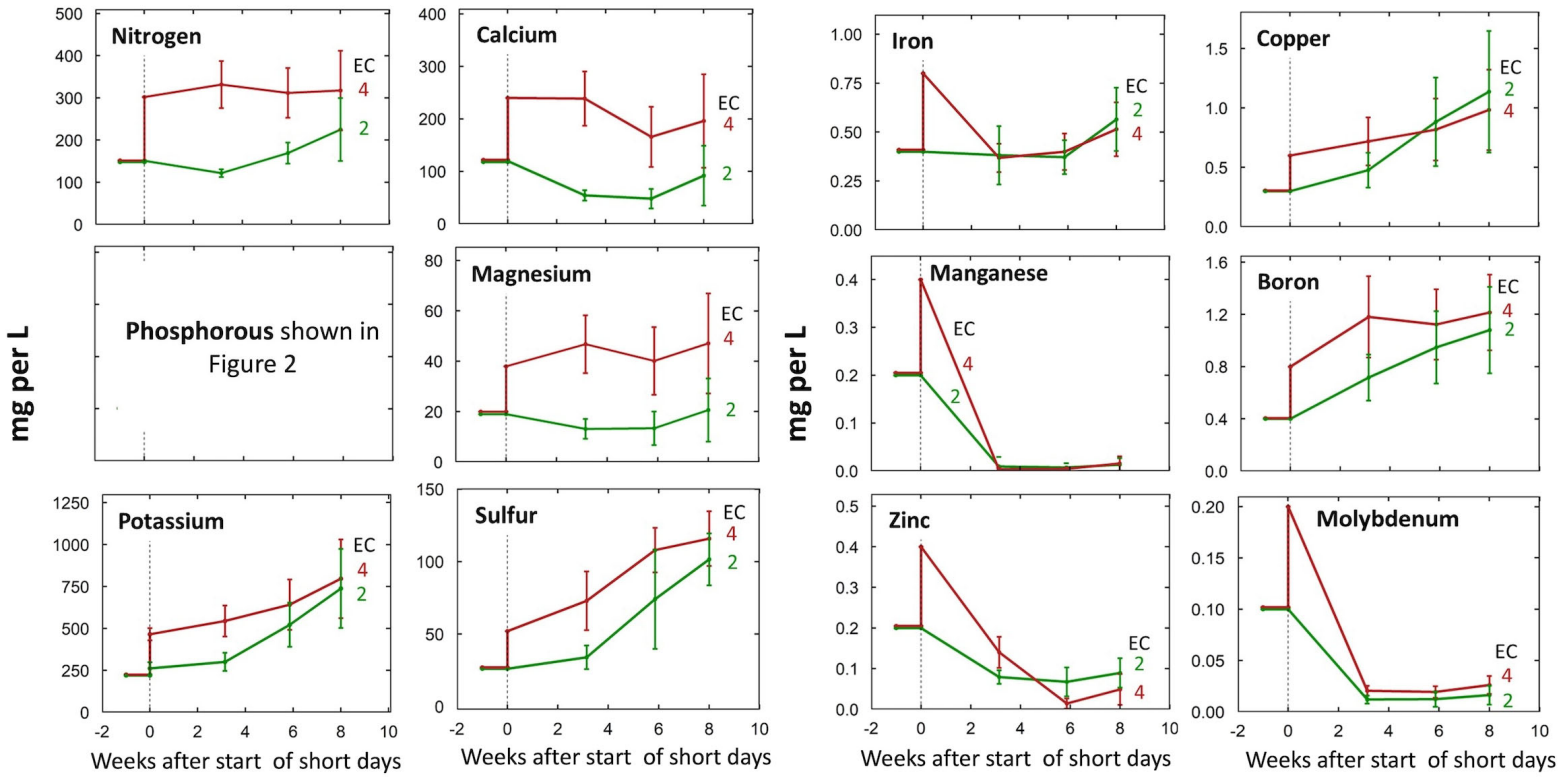
Change in individual nutrient solution concentrations over the eight-week life cycle at input EC’s of 2 or 4 mS per cm. All concentrations are shown in mg per L (ppm) of the element. Treatments were started at the transition to short days (week zero). Each data point represents the average of the three P treatments within each EC treatment (n = 3). Error bars represent standard deviation.

Unsurprisingly, solution concentrations of most ions were higher in the 4 mS per cm EC treatments (**Figure 1**), but we observed accumulations of S, K, B, and Cu across all treatments (**Figure 1**). Concentrations of Mn, Mo, and Zn in the root-zone solution were much lower than concentrations in the refill solution (**Figure 1**).

**Figure 2:**
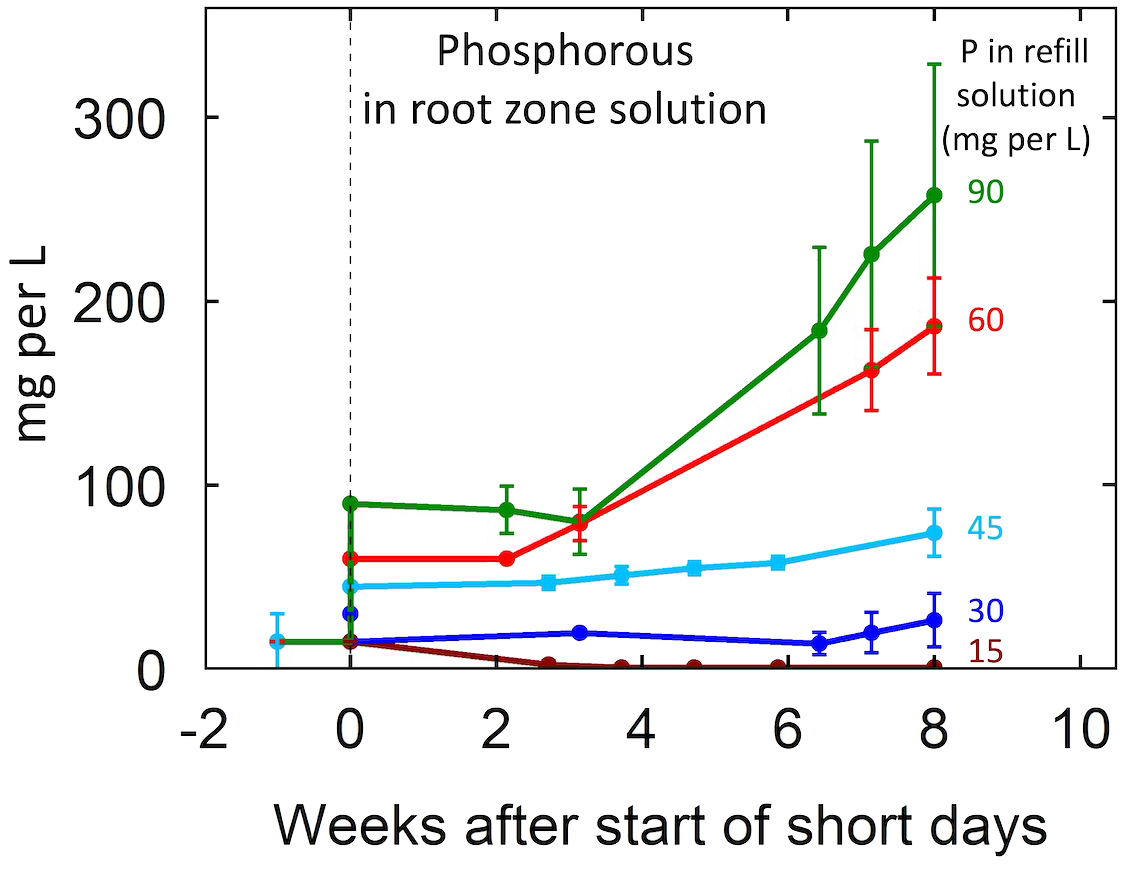
Change in the concentration of P over the eight-week life cycle for the five P levels in this study. The nutrient treatments were 15 mg per L during vegetative growth. Treatments commenced at the start of short days (week zero). The nutrient solution was recirculated (closed) so there was no leaching. The 15 mg per L treatment received 15 mg per L at each refill event (daily) but active uptake resulted in a low level in the closed circulating solution. The other concentrations provided P faster than the plants could absorb it and the solution concentration increased over time. Individual data points represent the mean of the two EC treatment levels within each P treatment. Error bars represent standard deviation from the mean (n = 2).

P inputs above 30 mg per L resulted in P accumulation in the root-zone in both EC treatments (**Figure 2**; **Supplementary Figure 4**). Notably, at all P levels, P accumulations were greater in the 2 mS per cm EC treatments than the 4 mS per cm EC treatments (data not shown). In the 2 mS per cm EC treatments, P inputs of 30, 60, and 90 mg per L resulted in solution P concentrations at harvest 44%, 242%, and 242% higher than the P input of the refill solution. In contrast, in the 4 mS per cm EC treatments P inputs of 30, 60, and 90 mg per L resulted in P concentrations only 25%, 180%, and 130% greater than P input.

### 3.3 Tissue nutrient concentrations and partitioning

**Figure 3:**
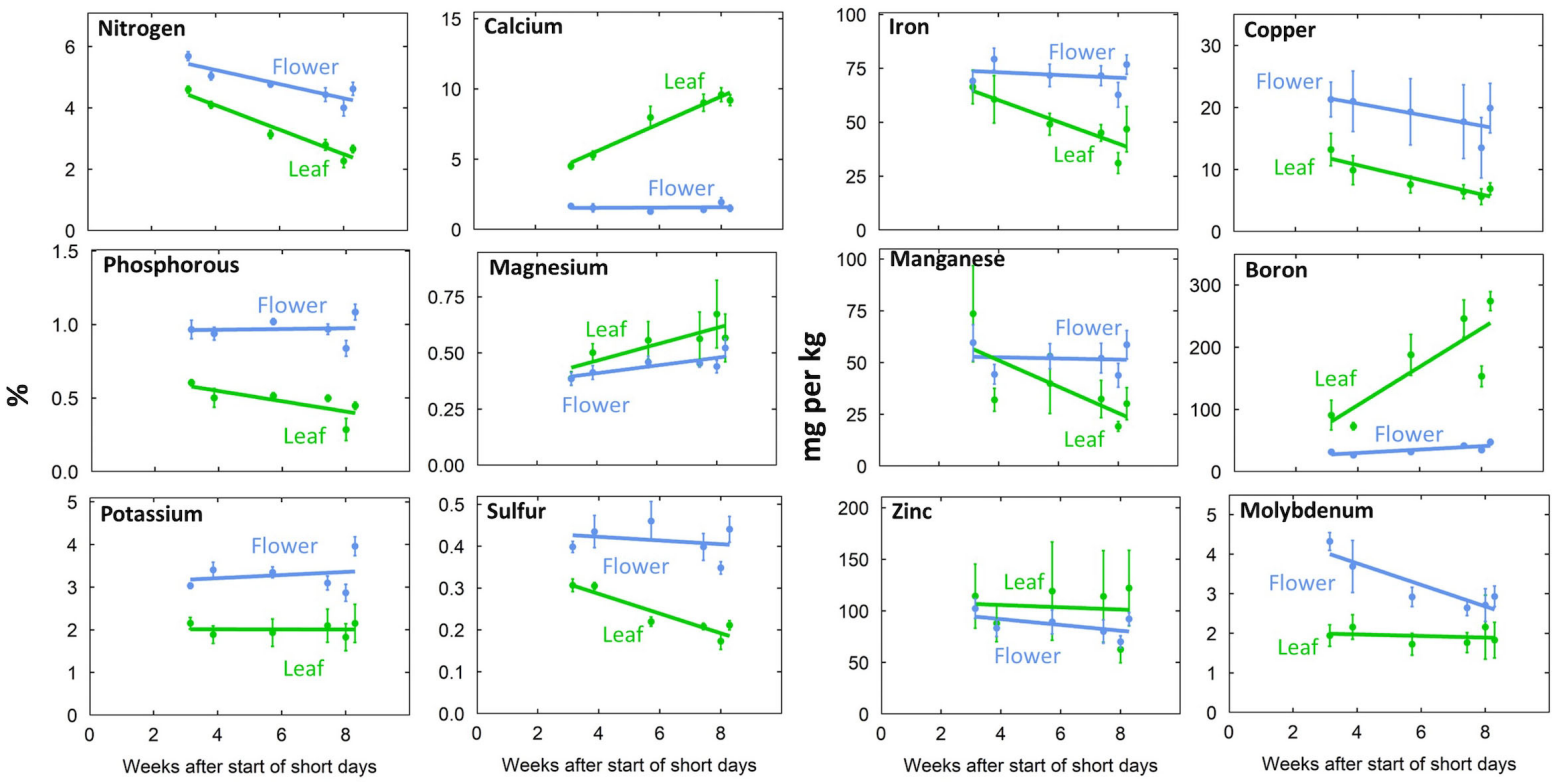
Time series of tissue concentration in leaf and flower tissue. Individual data points represent the mean of the two EC treatments and three P treatments (n = 6). Error bars represent standard deviation.

**Figure 4:**
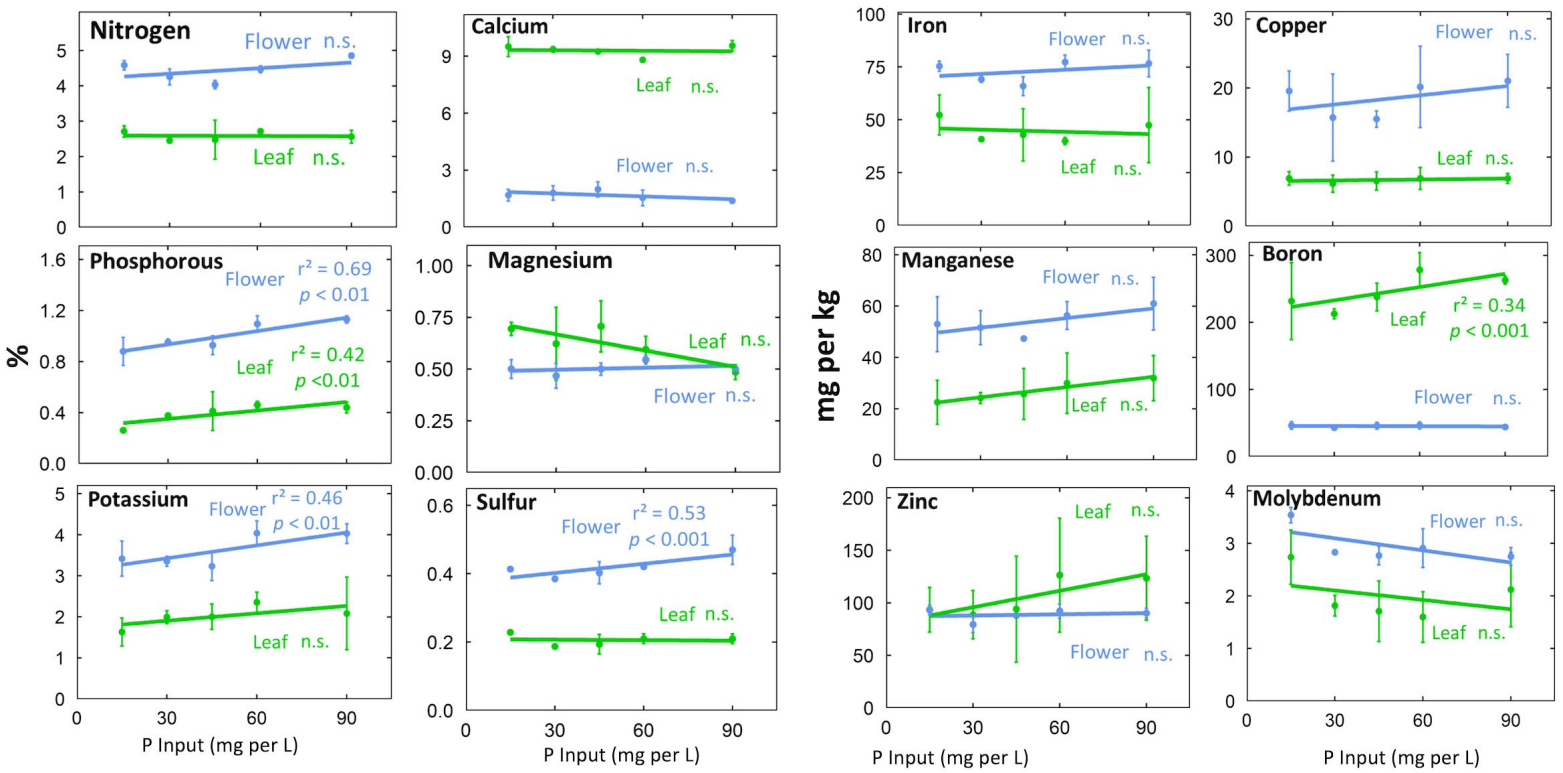
Effect of P concentration in the refill solution on tissue nutrient concentrations at harvest. Individual data points represent the mean of the two EC treatments (n=2) within each P treatment. Error bars represent standard deviation. The p-values represent the statistical significance of the relationship (slope of the line).

Leaf tissue nutrient concentrations (**Figures 3**, **4**) were within reported sufficiency ranges across all treatments throughout the duration of the study (Bryson and Mills, 2015; Cockson et al., 2020; Landis et al., 2019; Marschner, 2012).

The P input had significant effects on tissue P (p < 0.001). Increasing the P input from 15 to 90 mg per L doubled leaf P and increased flower P 70% at harvest (**Figure 4**). Tissue K typically increased with P input (**Figure 4**; p = 0.01). This is probably a result of additional K supplied by KH_2_PO_4_, which was used to supply P (**Supplementary Table 2**). Flower S (p <0.001) and leaf B (p <0.001) also increased with increasing P supply (**Figure 4**).

Generally, flowers had higher concentrations of mobile nutrients and leaves had higher concentrations of immobile nutrients (**Figures 3**, **4**; **Supplementary Table 3**). Flower N, Fe, Cu, P, Mn, K, S, and Mo concentrations were higher than leaf concentrations throughout the duration of flowering (**Figure 3**). At harvest, flower tissue concentrations of N, K, S, Fe, Mn, and Mo were roughly two-fold higher, and P and Cu concentrations were around three-fold higher than leaf concentrations in all treatments (**Figure 4**; **Supplementary Table 3**). This was calculated as the ratio of flower tissue concentration divided by leaf concentration, a dimensionless ratio. Surprisingly, tissue concentrations were not affected by the solution concentration (**Figure 4**).

## 4 Discussion

### 4.1 Effect of EC on yield and cannabinoid concentration

Doubling the nutrient solution concentration from an EC of 2 to 4 mS per cm had no effect on flower yield or cannabinoid concentration. Our findings are generally consistent with recent nutrition studies on cannabis in soilless media (Baas and Wijnen, 2023; Konvalina et al., 2024). In contrast, Velechovsky´ et al. (2024) reported THC yields increased 51% in a drain to waste system and 182% in a recirculating system when P, K, and Fe concentrations were elevated roughly 93%, 43%, and 940%, respectively. Cannabis may tolerate excessive nutrient concentrations in the root-zone, but it is not necessary for maximum yield or quality, and contributes to environmental pollution. These findings in hydroponics are relevant to soilless media root-zones that are continuously fertigated with a nutrient solution through drip irrigation. The goal of all systems in controlled environments should be to reduce the leaching of nutrients into the environment.

### 4.2 Effect of P on yield and cannabinoid concentration

Several studies have investigated the impact of P supply on yield quality of medical cannabis, but the reported optimal P was not consistent among studies. In this study, yield and quality did not increase above a P input of 15 mg per L. Westmoreland and Bugbee (2022) reported that maximum yield was achieved at 25 mg per L P, but lower P inputs were not investigated. Shiponi and Bernstein (2021b) investigated a wider range of 5 to 90 mg per L P and found that one cultivar achieved maximum yield at 30 mg per L P, but another cultivar had increasing yield up to 90 mg per L, however, yield only increased 20% with a three-fold increase in P. Using a central composite design, Bevan et al. (2021) predicted an optimal P input of 60 mg per L. In contrast, Cockson et al. (2020) reported that maximum yield and cannabinoid production were achieved with 11.25 mg per L P. The variation in optimal P between studies could be due to environmental differences like CO_2_ supplementation, light intensity, or temperature. Additionally, genetic variability could affect P requirements among cannabis cultivars (Hawkesford and Griffiths, 2019; Shenoy and Kalagudi, 2005; Shiponi and Bernstein, 2021b). Most studies indicate that a P supply less than 30 mg per L is adequate.

### 4.3 Nutrient accumulation in solution

The EC of the root-zone solution increased over time indicating that nutrient supply exceeded plant uptake. Phosphorus accumulated in the root-zone solution when P input exceeded 30 mg per L with greater accumulations at higher P inputs. This is consistent with the findings of P in leachate from Westmoreland and Bugbee (2022). Interestingly, P accumulation was not evident until two weeks after the induction of flowering, suggesting greater P uptake at the beginning of flowering. Generally, plant demand for external nutrient supply corresponds with the life stage (Jones et al., 2011). During the first few weeks after the start of short days cannabis exhibits a transitional period between the vegetative and reproductive stages marked by rapid stem elongation and the maturation of developing leaves, which could explain why P uptake remained high (Shiponi and Bernstein, 2021b). Nutrient uptake curves generated for sunflower (Heard and Park, 2008), lentil (Malhi et al., 2007), pea (Malhi et al., 2007), and small grains (Malhi et al., 2007) demonstrate greater nutrient accumulation during the vegetative and early bud formation with a pronounced decline in uptake from the root-zone at anthesis. Similarly, in poinsettia nitrogen uptake increases during the vegetative and inductive stages of flower development, but declines during anthesis (Whipker and Hammer, 1997).

Typically, P accumulations in the root-zone solution were greater in the 2 than in the 4 mS per cm EC treatments. In the 2 mS per cm EC treatments P inputs of 30, 60, and 90 mg per L resulted in solution P concentrations at harvest 44%, 242%, and 242% higher than the P input of the refill solution. In contrast, in the 4 mS per cm EC treatments P inputs of 30, 60, and 90 mg per L resulted in P concentrations only 25%, 180%, and 130% greater than P input. However, tissue P was similar between EC treatments. This suggests that P may have precipitated in the high EC treatments possibly as insoluble Ca(PO_4_)_2_. A precipitate was observed in tubs containing high EC treatments, but there was not a sufficient quantity for elemental analysis.

Despite low root-zone concentrations of Mn, Mo, and Zn in all treatments, tissue levels were within the sufficient range for metabolic processes. B, Cu, and S accumulated in the root-zone solution of all treatments, which was also observed by Savvas and Gizas (2002) in closed-system hydroponics. In the present study, there was no effect of high root-zone ion concentration on plant health or yield, but nutrient accumulation can cause imbalances and phytotoxicity (Lee et al., 1996; Savvas and Gizas, 2002). To manage nutrient accumulation and imbalances created by excessive fertilizer application, fertigation duration or frequency is often increased to flush accumulated salts from the media. In hydroponics, nutrient accumulations and imbalances are managed by periodically draining and replacing the root-zone solution. A better management practice is to avoid overfertilizing in the first place.

### 4.4 Nutrient partitioning in leaves and flowers

Nutrient distribution and remobilization within the plant is affected by nutrient supply, source-sink relations, life stage, and phloem mobility (Maillard et al., 2015; Ray et al., 2020). At harvest, mobile nutrient concentrations were higher in flowers and immobile nutrients were higher in leaves. This is consistent with the findings of Malík et al. (2025); Saloner and Bernstein (2021); Shiponi and Bernstein (2021b); Veazie et al. (2021); Velechovsky´ et al. (2024); Westmoreland and Bugbee (2022).

Cannabis accumulates significant P in flowers prior to harvest (Shiponi and Bernstein, 2021b; Veazie et al., 2021; Velechovsky´ et al., 2024; Westmoreland and Bugbee, 2022), and we found that flower P was 2.5 times higher than leaf P. Remobilization of P during reproductive development has been observed in other species. In rice, the developing panicle is the primary sink for P (Julia et al., 2016) and most grain P is remobilized from P stored in vegetative tissues. It has been suggested that P in cannabis flower is stored as phytic acid (Westmoreland and Bugbee, 2022). Time course data in soybean demonstrated increasing phytic acid accumulation during seed development (Raboy and Dickinson, 1987), but medical cannabis does not develop seeds. Further research is needed to elucidate the form of P stored in flowers.

In addition to P, flower Cu was 2.8 times higher than leaf Cu and flower S was 2 times higher than leaf S across all treatments. Cu accumulation in flowers has been observed in cannabis (Westmoreland and Bugbee, 2022), wheat (Garnett and Graham, 2005), and *Verbascum olympicum* (Güleryüz et al., 2006). In wheat, Cu is remobilized from leaves to developing grains post anthesis (Garnett and Graham, 2005). S accumulation in flower could be due to the accumulation of volatile S compounds found in cannabis flower, which increase with age (Oswald et al., 2021). Future research should explore the role of Cu and S in cannabis flower.

### 4.5 Potential effect of increased potassium in the increased phosphorous treatments

The principle of charge balance means that an increase in anions must be accompanied by an equal increase in cations. In this study we used potassium (K) to counterbalance the phosphate ions. This approach has been widely used in plant nutrition studies because there is a broad range optimal K concentrations in the rootzone and in plant tissue. The rootzone K concentration in the lowest P treatment was ample for maximum growth (**Supplementary Table 2**) and was ample in the leaves across all treatments (**Figure 4**). Indeed, the increased K on the rootzone did not increase K in the leaves, and only slightly increased K in the flowers from 3.5 to 4.1%. These concentrations are within the range for optimal growth.

### 4.6 On the lack of interaction between EC and P

There was no significant interaction between EC and P, which indicates that higher P levels are not needed even with a high background concentration of other nutrients. Similar findings were reported by Konvalina et al. (2024).

## 5 Conclusions

A refill EC of 2 mS per cm and P concentration of 15 mg per L were sufficient for maximum yield and cannabinoid production. Increasing EC from 2 to 4 mS per cm significantly increased nutrient accumulation in solution, but minimally affected tissue nutrient concentrations. P in solution accumulated to more than 300 mg per L at an input of 90 mg per L. Active uptake of P resulted in an average root-zone concentration of less than 0.1 mg per L at an input of 15 mg per L, which is typical of bioavailable P in agricultural soils. We conclude that cannabis tolerates high solution concentrations, but this does not improve yield or quality. These results are relevant to all types of root-zone environments, including peat-based media, coconut coir media and mineral wool substrates.

## Data Availability

The raw data supporting the conclusions of this article will be made available by the authors, without undue reservation.
