# Supplementary material for: Elevated root-zone P and nutrient concentration do not increase yield or cannabinoids in medical cannabis
Source: Front Plant Sci. 2025 Feb 20;16:1433985. doi: 10.3389/fpls.2025.1433985 (PMC11882401; doi:10.3389/fpls.2025.1433985)
Supplement: Supplementary file 1 [file DataSheet1.docx]

Supplementary Material

**Supplementary Table 1.** Nutrient solution composition at each EC treatment level.

| **Refill**  **solution EC**  **(mS per cm)** | | **N** | **K** | **Ca** | **Mg** | **S** | **Si** |  | **Fe** | **Mn** | **Zn** | **B** | **Cu** | **Mo** |
| --- | --- | --- | --- | --- | --- | --- | --- | --- | --- | --- | --- | --- | --- | --- |
| **2** | **mg**  **per L**  **mM** | 154  11 | 203  5.2 | 120  3 | 20  0.8 | 26  0.8 | 17  0.6 | **µM** | 1  18 | 0.2  3 | 0.4  3 | 0.4  40 | 1  16 | 0.01  0.1 |
| **4** | **mg**  **per L**  **mM** | 308  22 | 407  10.4 | 240  6 | 40  1.6 | 52  1.6 | 34  1.2 | **µM** | 2  36 | 0.4  6 | 0.8  6 | 0.8  80 | 2  32 | 0.02  0.2 |

**Supplementary Table 2.** P and K concentrations at each P treatment level. Values listed for K are representative of the base solution prior to the addition of KH_2_PO_4_ for P treatment.

| **P Treatment** |  | **P** | **K** |
| --- | --- | --- | --- |
| **15** | mg per L  mM | 15  0.5 | 19.6  0.5 |
| **30** | mg per L  mM | 30  1 | 39  1 |
| **45** | mg per L  mM | 45  1.5 | 58.7  1 |
| **60** | mg per L  mM | 60  2 | 78  2 |
| **90** | mg per L  mM | 90  3 | 121  3 |

**Supplementary Table 3.** The ratio of flower nutrient concentrations to leaf concentrations at harvest. A ratio greater than 1 indicates higher concentrations in flower tissue and is indicative of mobile nutrients. A ratio less than 1 indicates higher concentrations in leaves and is indicative of immobile nutrients. Values were similar among all EC and P treatments.

| **Ratio of flower to leaf tissue nutrient concentration** | | | | | | | | | | | | | |
| --- | --- | --- | --- | --- | --- | --- | --- | --- | --- | --- | --- | --- | --- |
|  | **EC** | **N** | **P** | **K** | **Ca** | **Mg** | **S** | **Fe** | **Mn** | **Zn** | **B** | **Cu** | **Mo** |
|  | **2** | 1.8 | 2.8 | 1.7 | 0.2 | 0.6 | 2.1 | 1.7 | 2.3 | 1.2 | 0.2 | 2.4 | 1.3 |
|  | **4** | 1.8 | 2.7 | 1.9 | 0.2 | 0.7 | 2 | 2 | 2 | 0.8 | 0.2 | 2.9 | 1.7 |


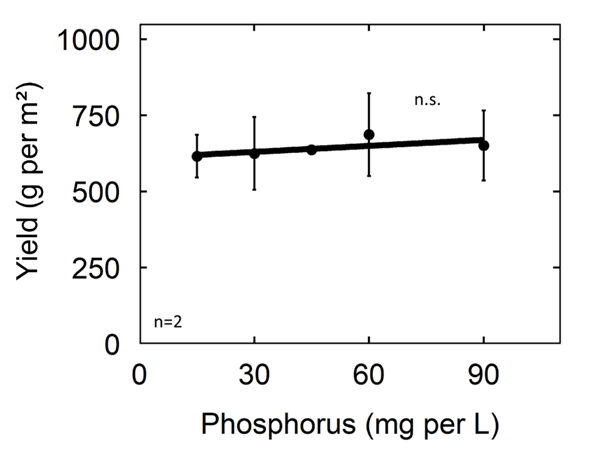


**Supplementary Figure 1.** Effect of P in the refill solution on flower yield at harvest. Each data point represents the mean of the two EC levels and error bars represent standard deviation. There was not a statistically significant effect of P in the refill solution (p = 0.95) or EC (p = 0.22) on dry flower yield.


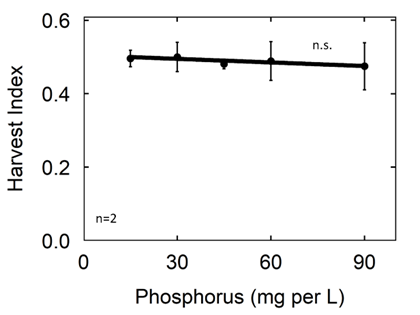


**Supplementary Figure 2.** Effect of P input on harvest index (HI). There was no significant treatment effect of EC on harvest index so data were pooled. Each data point represents the average of the two EC levels within each P treatment and error bars represent standard deviation.


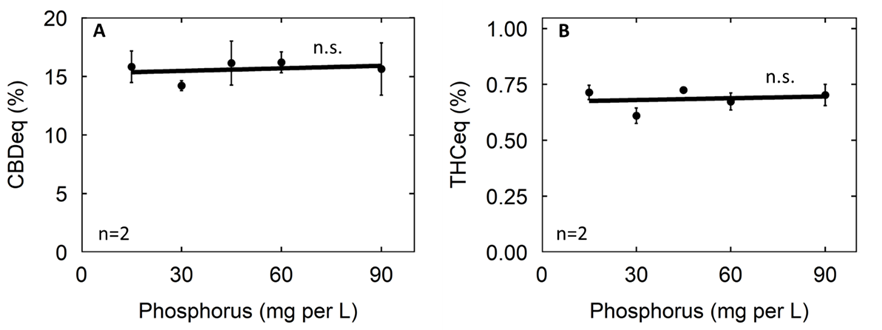


**Supplementary Figure 3.** Effect of P input on CBDeq (**A**) and THCeq (**B**) concentrations at harvest. Solution EC treatment did not have a significant effect on cannabinoid concentrations so data were pooled. Data points represent the average of the standard and high EC treatments and error bars represent the standard deviation.

**
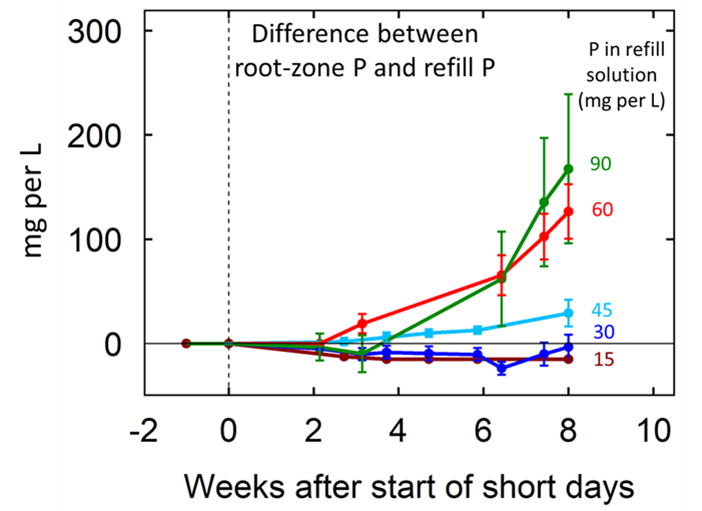
**

**Supplementary Figure 4.** The effects of P concentration in the refill solution on P accumulation in the recirculating solution over time. Individual data points represent the mean of the two EC treatment levels within each P treatment. Error bars represent standard deviation from the mean (n = 2).
